# Periodic Physical Disturbance: An Alternative Method for Controlling *Sitophilus zeamais* (Maize Weevil) Infestation

**DOI:** 10.3390/insects7040051

**Published:** 2016-09-29

**Authors:** Rashid Suleiman, Kurt A. Rosentrater, Bernard Chove

**Affiliations:** 1 Department of Agricultural and Biosystems Engineering, Iowa State University, Ames, IA 50011, USA; rashid@iastate.edu; 2 Department of Food Technology, Nutrition and Consumers Sciences, Sokoine University of Agriculture, Morogoro P.O. BOX. 3006, Tanzania; bchove06@yahoo.com

**Keywords:** *Sitophilus zeamais*, maize weevil, periodic physical disturbance, maize, insect mortality, stored products

## Abstract

*Sitophilus zeamais* Motschulsky is the most important insect pest of stored maize in tropical regions. The objective of this study was to determine the practicality of periodic physical disturbance on *S. zeamais* mortality and its adoption by smallholder farmers in developing countries. In this experiment, treatments and control were arranged in a randomized block design with three replications and three storage times in three regions of Tanzania. Region was used as the blocking variable. A total of 108 clean 20-L plastic containers were each loaded with 10 kg of fresh white dent corn and 0.50 kg of maize infested with *S. zeamais*. For the treatment, containers were disturbed twice a day, whereas for the controls the containers were not disturbed until the end of storage. The overall mortality rate (%) after 30, 60, and 90 days of storage were 88%, 96%, and 98%, respectively. A statistically significant difference (*p* < 0.05) was observed for the number of live *S. zeamais* between the control and experimental treatments. Additionally, the number of live *S. zeamais* in the treatment significantly decreased as storage time increased. This study shows the potential of a feasible, simple, affordable, and effective method of protecting maize grain for small-holder farmers in developing countries without using chemicals.

## 1. Introduction

Maize is the most important cereal and cash crop in sub-Saharan Africa (SSA) and is part of the staple diet for over 1.2 billion people in developing countries [1]. Current maize production in SSA is about 7 million metric tons [2], which is an increase of three percent from the 2012–2013 maize production year. Nevertheless, post-harvest losses (PHL) of cereal grain in SSA remain significantly higher (5%–40%) [3]. However, the exact magnitude of losses varies greatly from region to region and country to country and depends on several factors such as length of storage, drying and storage methods, storage structures, and pest damage [4]. In Tanzania, PHL of maize has been estimated to be between 15% and 26% [4]. The greatest portions of these losses occur in the field and during storage and are mainly due to insect infestation. The most economically important and widely occurring PHL insect pests of stored maize in Tanzania include *Sitophilus zeamais* Motschulsky, the maize weevil, and *Prostephanus truncatus* (Hons), the larger grain borer [5]. Preventing infestation from these pests remains a huge challenge for small-holder farmers in most countries in SSA, including Tanzania [6]. In addition, the problems have significantly increased in recent years due to the replacement of local varieties by improved varieties, which are mostly not pest resistant. This is increasing the demand for synthetic insecticides [7], which are commonly used to control insect pests of stored products [8,9]. However, inadequate education, haphazard application, lack of protective equipment, overuse, and lack of proper regulations of insecticides in developing countries [10] have resulted in a number of serious drawbacks, such as persistence in the environment, chemical residues in foodstuffs, and adverse health consequences to humans and animals [11,12]. Currently, national governments globally have set maximum residual levels (MRLs) for insecticides in food products including maize. Farmers are seeking alternatives to chemical insecticides to meet such demands. Physical control methods have been described as effective and alternative methods to pesticides to prevent and control pests during grain handling and storage [13].

Mechanical or physical techniques for control of stored-grain pests are based on the application of some kind of force or activities that manipulate the storage environment to provide conditions unfavorable to pests [14,15]. Physical control methods are not a new technique in grain protection and actually were the main techniques before synthetic insecticides came into use [14]. It is predicted that, in the near future, physical control methods will again be the predominant process in grain handling and storage [14,16] because of increased consumer awareness of the health risks of pesticide use and the demand for product-free synthetic insecticides. In addition, restrictions on the use of chemical insecticides such as methyl bromide are becoming more common. Physical control methods can be simple, affordable, and safe methods of controlling stored insect pests in grain facilities [16,17]. They include the use of techniques such as heat, cold, inert dust, aridity, physical exclusion of air, removal, and impact or physical disturbance [14].

A study conducted by Quentin et al. [18] examined the tumbling of beans in half-filled buckets every morning and evening, and found reduced *Acanthoscelides obtectus* (bean weevil) populations by 97% relative to controls without significant damage to the beans. A recent laboratory study which involved rolling coffee cans half-filled with maize one circumference twice a day reduced *S. zeamais* populations by 81% compared to the controls [19]. Similarly, Muir et al. [20] observed that, “during grain movement, insects infesting grain are subject to shaking, jarring, vibrations, and centrifugal forces which can be fatal to insects, and reduce grain temperatures to unfavorable levels for insect development.” In another study, conducted by Joffe and Clarke [21], shows rice weevils, *S. oryzae* (L.), are sensitive to pouring, and many insects were eliminated during the turning of the grain in a grain elevator. According to Joffe et al. [22], turning or physical disturbance of grain from one bin to another can significantly reduce live grain weevil infestation. The objective of this study was to determine the practicability of periodic physical disturbance on *S. zeamais*, maize weevil, mortality by subsistence farmers in developing countries as an alternative method to synthetic pesticides.

## 2. Materials and Methods 

### 2.1. Study Area

**Figure 1:**
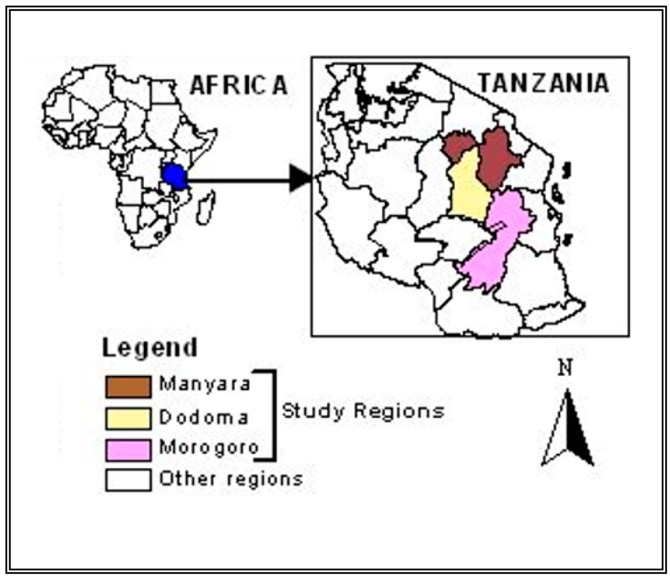
Map of Tanzania showing study regions.

This study was conducted in the maize-producing regions of Manyara, Dodoma, and Morogoro in Tanzania between November 2015 and February 2016 (Figure 1). These regions are each located in different agro-ecological zones (Northern, Central, and Eastern) and represent different patterns of maize production in the country. The Northern zone produces large quantities, the Central zone produces low quantities, and the Eastern produces moderate quantities of maize. All regions have a history of high post-harvest losses [4]. For each region, one major maize-producing district was purposely selected: Babati district representing the Manyara region; Chamwino district representing Dodoma region; and Kilosa district representing the Morogoro region (Figure 1). From each district, one ward was selected for the study. The wards selected are shown in Table 1. From each ward, three small-holder maize farmers were randomly chosen for this study. Each farmer was given twelve plastic containers—nine for treatments and three for control.

### 2.2. Experimental Design

This study employed a farmer participatory research approach and was developed in consultation with a statistician. This method attempted to incorporate farmers, agricultural extension officers, and researchers in the process. The study consisted of two trials: treatment and control. The experiment was conducted for three months in three districts from three different regions (Babati in the Manyara region, Chamwino in the Dodoma region, and Kilosa in the Morogoro region). A total of 108 clean 20-L (L 284 mm × W 234 mm × H 391 mm) plastic containers (36 per region) were used. Each container was loaded with 10 kg (about half capacity) of fresh white maize and 0.50 kg of white maize infested with mixed-aged adult *S. zeamais*. This quantity was chosen so that thorough physical disturbance (shaking 2–3 times for about 3 min) could readily be achieved by the farmers. If the containers were filled with more maize, some farmers had difficulty shaking the containers. The initial numbers of *S. zeamais* were determined (Table 2). To avoid asphyxiation of *S. zeamais* during storage, a small hole was drilled at the top of each container to allow airflow (this was not a hermetic storage study). All containers were sealed properly to avoid re-infestation. For the treatment, containers were disturbed twice a day (early in the morning and late in the evening), whereas the control containers were not disturbed until the end of the study. At the end of each storage time (30, 60, and 90 days), three treatment containers and one control from each farmer were randomly opened, then the number of live and dead *S. zeamais* were determined. As an additional aspect, grain damage was determined by visual observation at the end of each storage time, but was not measured.

### 2.3. Determination of Live and Dead Insects (Mortality Rate)

At the end of the first, second, and third months, two containers from each farmer were opened and poured onto a clean dry surface. After thorough mixing, to ensure insect and corn homogeneity, about one-fourth (2.5 kg) of the maize was randomly drawn from the mixture and then divided using a quartering technique to determine the number of live and dead *S. zeamais* by visual inspection [23]. The insect mortality rate (%) was calculated by using Equation (1) [24]

(1) $$\mathrm{Mortality}\;\left(\text{\%}\right)=\;\frac{\mathrm{Number}\;\mathrm{of}\;\mathrm{dead}\;\mathrm{insects}}{\mathrm{Total}\;\mathrm{number}\;\mathrm{of}\;\mathrm{insects}}\times 100$$

### 2.4. Data Analysis

The data collected (see supplementary material) were subjected to a one-way analysis of variance (ANOVA) using the Statistical Analysis System (SAS) software (version 9.4 for Windows; SAS Institute, Cary, NC, USA), with a general linear model PROG GLM (SAS Institute, 2011) using an α of 0.05, according to the blocking variable (region). If means were found to be significantly different (*p* < 0.05), Tukey’s HSD test was performed to determine where statistical differences among the means occurred. Multiple statistical comparisons were completed by pooling the data and using the Type III sums of square errors.

## 3. Results

### 3.1. Insect Mortality

**Figure 2:**
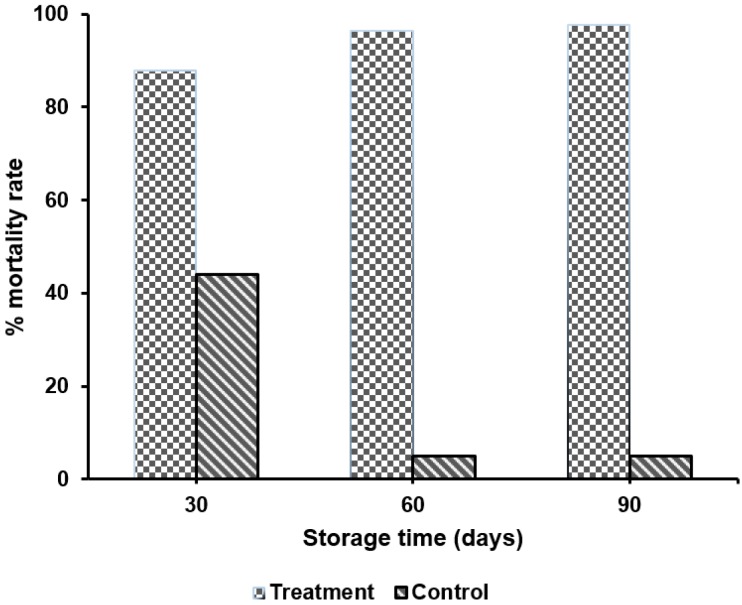
The overall effect of physical disturbance on *S. zeamais* mortality rate for treatment and control.

For all regions, statistical analysis (see supplementary material) indicated a significant difference (*p* < 0.05) between control and disturbed treatments for mortality rate of *S. zeamais* (Table 3). In other words, compared with the control, a significant increase of mortality rate for *S. zeamais* was observed in the disturbed treatments. Overall mortality rates of *S. zeamais* were determined by combining the mortality rates of all farmers in the same district. Overall mortality for the control is the average of three farmers at the end of each month. For the treatment, the overall mortality rate was obtained by taking an average of three containers from each farmer and a combined average of all three farmers in the same village. The differing errors vis-à-vis the treatment/region comparisons was accounted for by using the Type III sums of square errors. The results of overall mortality rates were 88% after the first month, 96% after the second month, and around 98% after the third month (Figure 2). Conversely, a declining trend in mortality rate was observed in the control treatments—mortality rate in the control treatment was less than 50% (Table 3). This was expected, as the control containers were not disturbed, and the insects were allowed to naturally grow and propagate. Moreover, it could have been the end of the life cycle of *S. zeamais,* as life cycle of *S. zeamais* is around 36 days. When examining experimental treatments for each of the districts, the mortality rate for the Chamwino district (Dodoma region) increased from 91% in the first month to 99% in the third month for the disturbed treatment. For the Morogoro region (Kilosa district), mortality rate of *S. zeamais* after 30, 60, and 90 days were 96%, 89%, and 98%, respectively (Table 3); the mortality rate in the second month was slightly lower than in the first and third month. Moreover, no significant difference (*p* < 0.05) was observed in the mortality rate in the Manyara region; all mortality levels were above 97%. Overall, it appears that the experimental treatments were all nearly 100% effective in all districts, for all three months of the study.

### 3.2. Number of Live Insects

Table 4 indicates the number of live insects throughout the study. The results show a significant difference (*p* < 0.05) in the number of live insects among the control treatments—in other words, the insect population was growing. However, no significant differences (*p* < 0.05) were observed for the disturbed treatments (Table 4)—because the insects kept dying at an increasing rate. The number of live *S. zeamais* in the control treatments increased significantly with storage time for all study sites in the three regions. As expected, a high number of *S. zeamais* was found at the end of storage time (90) days for the unturned control samples. In fact, the number of live *S. zeamais* almost tripled in the second month and quadrupled in the third month of the study. On the other hand, for the disturbed treatments, the number of live insects decreased as the storage time increased. For instance, the number of live adult *S. zeamais* in Dodoma region was 10, 2, and 0 after 30, 60, and 90 days of storage, respectively. Additionally, the results show that at the end of the study (90 days) there were no live insects in any of the three regions (Table 4) for the disturbed treatments. The number of live *S. zeamais* was related to the mortality rate in the disturbed treatments.

## 4. Discussion

The high mortality rate and the low populations of *S. zeamais* in the disturbed treatments were due to the physical disturbance of the containers. Physical disturbance has been previously studied as an alternative method to reduce insect infestations in stored grain [14,17,18,19,22,25,26,27,28,29,30,31]. The method is known to significantly reduce insect populations in all stages of development (from eggs, larvae, and pupae to adults) due to mechanical agitation of the grain [14,31,32]. According to [26], physically disturbing the grain at least two or more times a week may significantly prevent insect development and reduce grain infestations. Although the actual reason for insect death has not been identified in this study, it is surmised that death occurred because the insects were not be able to completely bore holes into the maize kernels (to feed or lay eggs), and the frequent disturbance ultimately resulted in the insects either starving or reaching the end of their lifespan. This study confirmed that almost all *S. zeamais* were dead (98% mortality rate) at the end of the study. Moreover, no grain damage from insect infestation was observed in any of the three regions after 30, 60, and 90 days of storage for the disturbed treatments. This supports the supposition that, by disturbing the insects, their feeding behavior and egg deposition ability was interrupted. Some grain damage in control containers was observed (but not quantified) as the insects could bore holes into the kernels, eat the grain, and produce byproduct chaff from their activities.

According to [31], turning of grain kills the insects outside of the grains as well as those inside the grains. The mortality rates of *S. zeamais* in the third months were 99%, 98%, and 100% in Dodoma, Morogoro, and Manyara regions, respectively (Table 3). These results were consistent with the findings of other previous studies. For instance, one study found 96% mortality for adults *S. oryzae* when small wheat sacks were dropped several times a day [28]. Furthermore, visual observation of the maize in this study found minimum damage. A similar finding was reported by [18]. This could be one of the reasons for low adult emergence in the turning treatments. Many studies reported mechanical damage as the most important factor in grain storability because kernel damage facilitates insects and fungal invasion [33].

It should be noted that moderate levels of insect mortality were observed in the control treatments at the end of the first month (30 days) for the Morogoro and Manyara regions. Since mixed age *S. zeamais* were introduced into all maize samples at the beginning of the study, these mortality rates observed at 30 days most likely were a manifestation of the end of the life cycle of *S. zeamais*. Although the death rate was moderately high for these regions, the total number of insects increased. In fact, a number of live insects for all control treatments increased over time. According to [34], the average life cycle of *S. zeamais* from egg to adult is about 36 days. Likewise, the numbers of live *S. zeamais* in the disturbed treatments decreased with time (Table 4), and mortality rates were dramatically high.

## 5. Conclusions

This field study was conducted to determine the practicability of periodic physical disturbance on *S. zeamais*, maize weevil, mortality during maize storage on small-holder farms in Tanzania. Physical disturbance was a very effective and potentially feasible method for protecting maize grain from *S. zeamais* infestation and can thus dramatically increase maize storage for either food, feed, or seed uses. Results have shown that containers disturbed (shaken) twice per day can significantly reduce *S. zeamais* infestation and will result in no damage to the maize kernels. After three months of storage, the mortality of *S. zeamais* amongst all districts was 98%, and there were no live *S. zeamais* in any container in any of the three regions. Hence, this study demonstrates the potential of a simple, affordable, feasible, safe, and effective method of protecting maize grain for small-holder farmers in developing countries who cannot afford modern and costly methods to control maize grain from insect infestation. This approach is a possible solution to reduce maize damage and infestation problems without using chemicals. Future work should aim to implement this approach in other regions in sub-Saharan Africa to test the efficacy and adoption potential. Additionally, training, extension, and outreach activities could help promote this approach to grain storage on small farms.

## Figures and Tables

**Table 1:** Sampling plan for physical disturbance study.

Region	District	Ward	Village	Number of Farmers	Number of Containers
Dodoma	Chamwino	Ikawa	Makoja	3	36
Morogoro	Kilosa	Mabwerebwere	Muungano	3	36
Manyara	Babati	Gallapo	Gallapo Mjini	1	12
			Gallapo Kati	1	12
			Chalo B	1	12

**Table 2:** Initial numbers of *S. zeamais* in each region per 0.5 kg of infested maize.

Storage Time (days)	Initial Number of *S. zeamais*					
	Dodoma		Morogoro		Manyara	
	Control	Disturbed	Control	Disturbed	Control	Disturbed
30	89	53	28	21	75	30
60	52	54	25	27	73	41
90	74	51	23	20	120	86

**Table 3:** Effect of physical disturbance on mortality rate (%) of *S. zeamais*.

Storage Time (days)	Control			Treatment		
	Dodoma	Morogoro	Manyara	Dodoma	Morogoro	Manyara
30	10 ± 12 ^a^	43 ± 13 ^a^	32 ± 9 ^a^	91 ± 4 ^a^	96 ± 4 ^a^	98 ± 4 ^a^
60	8 ± 2 ^b^	24 ± 17 ^b^	6 ± 1 ^b^	95 ± 1 ^a^	89 ± 6 ^b^	100 ± 1 ^a^
90	6 ± 5 ^b^	21 ± 11 ^b^	10 ± 17 ^b^	99 ± 1 ^a^	98 ± 3 ^a^	100 ± 0 ^a^

Each value inside the table is the mean ± standard deviation of three replicates. Means followed by the same letter in a single column indicate no significant difference (*p* < 0.05, *n* = 18 per region per treatment).

**Table 4:** Number of live *S. zeamais* for the control and disturbed treatments after 30, 60, and 90 days.

Storage Time (days)	Control (stationary)			Disturbed (shaken)		
	Dodoma	Morogoro	Manyara	Dodoma	Morogoro	Manyara
30	20 ± 8 ^c^	9 ± 2 ^c^	12 ± 4 ^c^	10 ± 2 ^a^	2 ± 1 ^a^	3 ± 1 ^a^
60	68 ± 31 ^b^	49 ± 35 ^b^	77 ± 44 ^b^	2 ± 1 ^b^	5 ± 1 ^a^	0 ± 0 ^a^
90	109 ± 22 ^a^	119 ± 35 ^a^	152 ± 36 ^a^	0 ± 0 ^b^	0 ± 0 ^a^	0 ± 0 ^a^

Each value inside the table is the mean ± standard deviation of three replicates. Means followed by the same letter in a single column indicate no significant difference (*p <* 0.05, *n* = 18 per region per treatment).

## Supplementary Materials

The supplementary material are available online at www.mdpi.com/2075-4450/7/3/47/s1.

## References

[1] IITA International Institute of Tropical Agriculture (IITA)—Maize (*Zea mays*) Crop, 2016. http://www.iita.org/maize.

[2] Food and Agriculture Organization of the United Nations Statistics Division (FAO) Maize Production in Sub-Saharan Africa 2013/2014 Production Year, 2014. http://faostat3.fao.org/browse/Q/QC/E.

[3] World Bank (2011). Missing Food: The Case of Postharvest Grain Losses in Sub-Saharan Africa.

[4] Africa Postharvest Losses Information System (APHLIS) Losses Tables; Estimated Postharvest Losses (%), 2014. http://www.aphlis.net/?form=home.

[5] Rugumamu C.P. (2012). Assessment of post-harvest technologies and gender relations in maize loss reduction in Pangawe village eastern Tanzania. Tanzania J. Sci..

[6] Suleiman R., Rosentrater K.A., Bern C.J. (2015). Evaluation of maize weevils *Sitophilus zeamais* Motschulsky infestation on seven varieties of maize. J. Stored Prod. Res..

[7] Demissie G., Teshom A., Abakemal D., Tadesse A. (2008). Cooking oils and “Triplex” in the control of *Sitophilus zeamais* Motschulsky (Coleoptera: Curculionidae) in farm-stored maize. J. Stored Prod. Res..

[8] Dal Bello G., Padin S., Lastra C.L., Fabrizio M. (2000). Laboratory evaluation of chemical-biological control of the rice weevil (*Sitophilus oryzae* L.) in stored grains. J. Stored Prod. Res..

[9] Nwosu L.C., Adedire C.O., Ogunwolu E.O., Ashamo M.O. (2015). Relative susceptibility of 20 elite maize varieties to infestation and damage by the maize weevil, *Sitophilus zeamais* (Coleoptera: Curculionidae). Int. J. Trop. Insect Sci..

[10] Wilson C., Tisdell C. (2004). Economics, Ecology and the Environment. Why Farmers Continue to Use Pesticides Despite Environmental, Health and Sustainability Cost. http://espace.library.uq.edu.au/view/UQ:154961/WP53.pdf.

[11] Khan A.R., Selman B.J. (1989). *Nosema* spp. (Microspora: Microsporida: Nosematidae) of stored-product Coleoptera and their potential as microbial control agents. Agric. Zool. Rev..

[12] Ngowi A.V.F., Mbise T.J., Ijani A.S.M., London L., Ajayi O.C. (2007). Smallholder vegetable farmers in Northern Tanzania: Pesticides use practices, perceptions, and cost and health effects. Crop Prot..

[13] Jayaprakash S.A., Mohan S., Ramaraju K. Egg removal device for the management of three stored product pests. Proceedings of the 10th International Working Conference on Stored Product Protection.

[14] Banks H.J. Impact, physical removal and exclusion for insect control in stored products. Proceedings of the 4th International Work Conference Store Products Protection.

[15] Paliwal J., Jayas D.S., White N.D.G., Muir W.E. (1999). Effect of pneumatic conveying of wheat on mortality of insects. Appl. Eng. Agric..

[16] White N.D., Jayas D.S., Demianyk C.J. (1997). Movement of grain to control stored-product insects and mites. Phytoprotection.

[17] Facknath S. (1993). Effect of grain tumbling on infestation by some insect pests. Rev. Agric. Sucr. Maurice.

[18] Quentin M.E., Spencer J.L., Miller J.R. (1991). Bean tumbling as a control measure for the common bean weevil, *Acanthoscelides obtectus*. Entomol. Exp. Et Appl..

[19] Bbosa D. (2014). Pesticide Free Methods of Maize Weevil Control in Stored Maize for Developing Countries. Chapter 4: Effect of Storage Container Physical Disturbance on Maize Weevil Mortality. Master’s Thesis.

[20] Muir W.E., Yacuik G., Sinha R.N. (1977). Effects of temperature and insect and mite population of turning and transferring farm-stored wheat. Can. Agric. Eng..

[21] Joffe A., Clarke B. (1963). The effect of physical disturbance or turning of stored maize on the development of insect infestations-II. Laboratory studies with *Sitophilus oryzae* (L.). S. Afr. J. Sci..

[22] Joffe A. (1963). The effect of physical disturbance or turning of stored maize on the development of insect infestation-I. Grain elevation studies. S. Afr. J. Sci..

[23] Schuler N.J., Bern C.J., Loy D.D., Brumm T.J., Strohbehn D.R. (2014). Mixing beef feed rotations containing distillers’ wet grains. Appl. Eng. Agric..

[24] Omotoso O.T., Oso A.A. (2005). Insecticidal and insect productivity reduction capacities of *Aloe vera* and *Bryophyllum pinnatum* on *Tribolium castaneum* (Herbst). Afr. J. Appl. Zool. Environ. Bio..

[25] Bailey S.W. (1962). The effects of percussion on insect pests of grain. J. Econ. Entomol..

[26] Bailey S.W. (1969). The effects of physical stress in the grain weevil, *Sitophilus granarius*. J. Stored Prod. Res..

[27] Bryan J.M., Elvidge J. (1977). Mortality of adult grain beetles in sample delivery systems used in terminal grain elevators. Can. Entomol..

[28] Loschiav S.R. (1978). Effect of disturbance of wheat on four species of stored-product insects. J. Econ. Entomol..

[29] Ungsunantwiwat A., Mills R.B. (1979). Influence of medium and physical disturbances during rearing on development and numbers of *Sitophilus progeny*. J. Stored Prod. Res..

[30] Plarre R., Reichmuth F. (2000). Impact. Alternatives to Pesticides in Stored-Product IPM.

[31] Facknath S. (2006). Combination of neem and physical disturbance for the control of four insect pests of stored products. Int. J. Trop. Insect Sci..

[32] Bahr I. Reduction of stored product insects during pneumatic unloading of ship cargoes. Proceedings of the 5th International Working Conference on Stored Products Protection.

[33] Ng H.F., Wilcke W.F., Morey R.V., Meronuck R.A., Lang J.P. (1998). Mechanical damage and corn storability. Trans. ASAE.

[34] Sharifi S., Mills R.B. (1971). Radiographic studies of *Sitophilus zeamais* mots. In wheat kernels. J. Stored Prod. Res..

